# The quantification and role of diffuse myocardial fibrosis in familial dilated cardiomyopathy - an equilibrium contrast cmr study

**DOI:** 10.1186/1532-429X-13-S1-O108

**Published:** 2011-02-02

**Authors:** Daniel M Sado, Andrew S Flett, Christopher M Cook, Caroline J Coats, Giovanni Quarta, Jonathan M Hasleton, Derek J Hausenloy, Perry M Elliott, James C Moon

**Affiliations:** 1 The Heart Hospital, London, UK

## Objective

To evaluate the role of diffuse myocardial fibrosis (DMF) in familial Dilated Cardiomyopathy (fDCM) using Equilibrium Contrast-CMR (EQ-CMR).

## Background

Post mortem/biopsy studies have shown that DMF occurs in DCM, but its clinical significance remains largely unknown as its assessment requires invasive biopsy. EQ-CMR is a recently described technique that permits the non-invasive and accurate assessment of DMF by measuring the myocardial contrast volume of distribution (Vd(m)) at equilibrium contrast [1].

## Methods

Using international diagnostic guidelines we identified 28 patients with fDCM (mean age 42, 61% male, 89% NYHA class I, 11% NYHA class II). This cohort excluded the most severe cases of fDCM due to the high prevalence of permanent pacemakers or ICDs.

The Vd(m) (a measure of DMF) was compared to that obtained in healthy volunteers (mean age 35, 63% male) and also to other indexed CMR parameters, including left atrial area and echocardiographic parameters of diastolic function (tissue doppler and strain imaging).

## Results

fDCM patients had increased LV size (95mls/m2 vs 73mls/m2 , p=0.0001), worse ejection fraction (55% vs 69%, p<0.0001), increased indexed left atrial area (LAAi) (12.3cm2/m2 vs 9.5cm2/m2, p=0.0004) when compared to healthy volunteers. Significantly impaired diastolic function was detected in 14% of fDCM patients. No patients had worse than mild mitral regurgitation.

**Figure 1:**
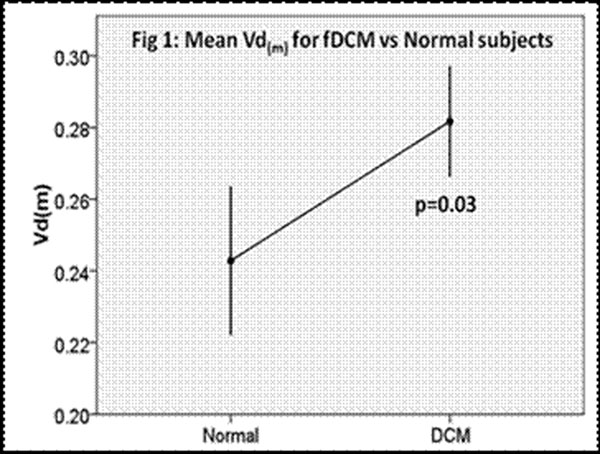


**Figure 2:**
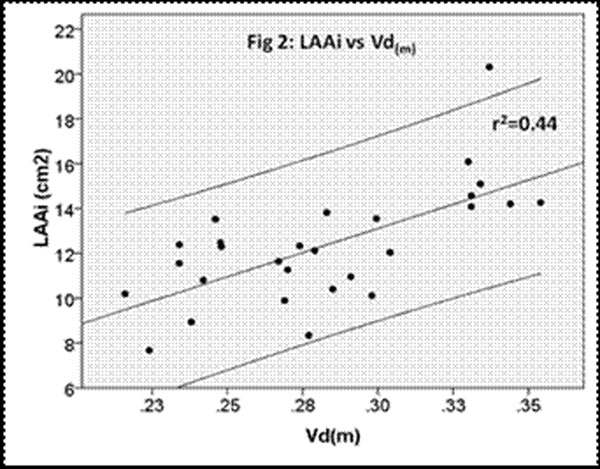


The Vd(m) in patients was significantly greater than that of healthy volunteers (Fig 1) (0.282 vs 0.234, p=0.003). Vd(m) correlated with LAAi (fig 2) (r2= 0.44, p<0.0001) and EF (r2=0.12, p=0.034), but was independent of indexed LV diastolic volume, mass and echocardiographic derived parameters of diastolic function.

On univariate analysis, LAAi was predicted by both Vd(m) (r2= 0.44) and E/Ea (r2= 0.31), which on multivariate analysis were independent with Vd(m) being the strongest predictor.

## Conclusion

EQ-CMR can reliably and non-invasively measure increased Vd(m), reflecting DMF even in patients with mild fDCM. Diffuse myocardial fibrosis in this tightly defined fDCM cohort correlates with known prognostic variables, particularly LA size and EF. For LA size, DMF is independent of diastolic function and the most important predictor. As such, these data suggest EQ-CMR is measuring a key disease parameter. Further study is recommended.

## References

[B1] Circulation 2010 122 2 138 144 10.1161/CIRCULATIONAHA.109.930636 20585010

